# Multi-label classification of retinal disease via a novel vision transformer model

**DOI:** 10.3389/fnins.2023.1290803

**Published:** 2024-01-08

**Authors:** Dong Wang, Jian Lian, Wanzhen Jiao

**Affiliations:** ^1^School of Information Science and Electrical Engineering, Shandong Jiaotong University, Jinan, China; ^2^School of Intelligence Engineering, Shandong Management University, Jinan, China; ^3^Department of Ophthalmology, Shandong Provincial Hospital Affiliated to Shandong First Medical University, Jinan, China

**Keywords:** retinal image, deep learning, multi-label classification, machine vision, medical image analysis

## Abstract

**Introduction:**

The precise identification of retinal disorders is of utmost importance in the prevention of both temporary and permanent visual impairment. Prior research has yielded encouraging results in the classification of retinal images pertaining to a specific retinal condition. In clinical practice, it is not uncommon for a single patient to present with multiple retinal disorders concurrently. Hence, the task of classifying retinal images into multiple labels remains a significant obstacle for existing methodologies, but its successful accomplishment would yield valuable insights into a diverse array of situations simultaneously.

**Methods:**

This study presents a novel vision transformer architecture called retinal ViT, which incorporates the self-attention mechanism into the field of medical image analysis. To note that this study supposed to prove that the transformer-based models can achieve competitive performance comparing with the CNN-based models, hence the convolutional modules have been eliminated from the proposed model. The suggested model concludes with a multi-label classifier that utilizes a feed-forward network architecture. This classifier consists of two layers and employs a sigmoid activation function.

**Results and discussion:**

The experimental findings provide evidence of the improved performance exhibited by the suggested model when compared to state-of-the-art approaches such as ResNet, VGG, DenseNet, and MobileNet, on the publicly available dataset ODIR-2019, and the proposed approach has outperformed the state-of-the-art algorithms in terms of Kappa, F1 score, AUC, and AVG.

## 1 Introduction

The retina, as a fundamental component of the ocular system, plays a crucial role in facilitating human visual function. The retina is situated at the posterior region of the eye and plays a crucial role in converting incoming light into electrical impulses. These signals are subsequently transmitted by the optic nerve to the brain (Yokomizo et al., 2019). Based on the inherent characteristics of the retina, it possesses the capacity to serve as an indicator for ocular ailments as well as many physiological conditions, including but not limited to diabetes and neurological disorders (Montesano et al., 2021; Zhou et al., 2021).

Taking advantage of fundus retina imaging evaluation can reveal many retinal illnesses, such as diabetes retinopathy (DR), glaucoma, and age-related macular degeneration (AMD). It is important to acknowledge that a significant number of individuals residing in Asian countries such as China and India are experiencing the adverse effects of DR (Ayoub et al., 2022). In the field of ophthalmology, glaucoma has emerged as a prevalent cause of enduring visual impairment (Mokhles et al., 2017; Sun et al., 2022). According to Schmitz-Valckenberg et al. (2016), AMD is widely acknowledged as the primary cause of complete vision impairment among individuals aged 50 and beyond. The precise identification of retinal lesions has the potential to enhance the timely detection and subsequent treatment of ocular illnesses. Early detection of retinal lesions has the potential to delay the progression of visual impairment resulting from degenerative disorders. Consequently, early diagnosis can also contribute to the advantageous outcomes of quick treatment.

Automatic machine vision-aided diagnosis system has attracted broadly attention from both clinical and academic fields (Abràmoff et al., 2010). It can mitigate the burden of ophthalmologists by avoiding the time-consuming, labor-tedious, and error-prone manual inspections. In addition, the employment of automated retinal image analysis can further eliminate the variability of image interpretation even when there are insufficient number of specialists of retinal image analysis (Mokhashi et al., 2021). Before the powerful deep learning methods have been proposed, a large number of machine learning-based retinal image analysis algorithms have been exploited in this area. As an early work of branch retinal vein occlusion (BRVO), Chen et al. (2014) proposed the hierarchical local binary pattern (LBP) to represent the characteristics of the fundus image. A BRVO dataset was constructed, and the comparison experiments were conducted using the images in this dataset. In the work of retinal image classification (Kumudham, 2015), Kumudham used the LBP features extracted from the hard exudate regions in retinal images and a support vector machine (SVM) classifier. Accordingly, each retinal image can be classified into normal and abnormal cases for diabetic macular edema (DME). Kothare and Malpe (2019) proposed an empirical framework consisting of requisite number of images and a group of methods to predict the possibility of DR. These methods include SVM and naive Bayes (NB) as the classifiers as well as the LBP for feature extraction. To discriminate the presence of DR and grade the severity of DR in retinal images without lesion segmentation, Berbar (2022) first employed the pre-processing techniques, including histogram matching and median filter, to the green channels of retinal images. Then, the contrast-limited adaptive histogram equalization was leveraged as well as the unsharp filter, to note that each image was segmented into small patches, from which the LBP features were generated. In addition, an SVM was taken as the classifier to implement the retinal image classification. In general, the study of Berbar (2022) can grade the severity of DR into three different levels. Recently, the study of Reddy and Ravindran (2022) presented an automatic screening platform to recognize DR in retinal images. The proposed classification scheme consists of two phases. In the first step, the retinal images were divided into four regions, namely, hard exudate, microaneursym, hemorrhage, and cotton wool spot. Second, three classifiers, such as k-nearest neighbor (KNN), gaussian mixture model (GMM), and SVM, were exploited to realize retinal image classification and DR severity grading. The classical machine learning methods rely heavily on the manually designed features extracted from the retinal images and an appropriate classifier. However, according to the complicated characteristics of the retinal images and the variation of illuminations, it remains a challenge to determine the optimal set of feature and the parameters of one classifier in a manual fashion.

On the other hand, the deep learning-based architectures have achieved more promising outcomes than the machine learning techniques. After the early study in 2016 from Google for classification of DR in fundus photographs, Hunt et al. (2020) presented a low-shot, self-supervised deep learning method for classification of retinal fundus images. The low-shot mechanism of learning in this study greatly resolved the problem of insufficient image samples, which is a major obstacle in most of the deep learning applications. To implement the detection of DR at its early stage, the study Meshram et al. (2021) proposed an investigation of the applications of deep learning models for retinal image classification. In general, the deep learning architectures, including the conventional convolutional neural network (CNN) and deep CNNs, were incorporated in this survey. In the study of Tak et al. (2021), a deep CNN model was trained to classify between different categories of AMD images. Accordingly, 420 wide-field retinal images were included in the training process for classifying the exudative and non-exudative AMD cases, and the accuracy achieved by the proposed CNN model is 88%. Umamageswari et al. (2022) provided an approach to identify exudates and veins with retinal images for the diagnosis of diabetics. Specifically, a CNN was proposed for retinal image recognition. Recently, to segment and classify the retinal images in a unified way, Kumari et al. (2023) proposed an efficient CNN model. To be specific, the input images for the proposed model were pre-processed using the green channel images, histogram-based algorithms, and noise elimination techniques. The features were extracted from the segmented images using the watershed algorithm as well as principal component analysis (PCA) technique, to note that the publicly available datasets used in this study were DRIVE (Asad et al., 2014), STARE (Guo, 2020), and CHASE DB1 (Yu et al., 2019). Most of the deep learning-based methods currently depend on the convolutional modules leveraged to extract the image embeddings for accurate classification.

Note that the above-mentioned approaches were originally designed for single-label classification of retinal images. However, there are usually more than one type of lesions appeared in practical scenarios. In addition, the simultaneous understanding of multiple lesions in an retinal image could provide more information from the associations between various diseased areas. Therefore, multi-label classification of retinal image has also be paid attention by a variety of machine vision and deep learning algorithms. Omar et al. (2017) presented a multi-label learning model to implement the exudate lesion classification based on the multi-scale LBP features. Sequentially, the KNN, neural network radial base function (NN-RBF), and neural network back-propagation (NN-BP) were taken as classifiers. With the employment of deep learning, the study of Prawira et al. (2021) used both the AlexNet (Krizhevsky et al., 2012) and VGG16 (Simonyan and Zisserman, 2014) models to deal with the task of multi-label retinal image classification. In total, there are three types of lesions, including DR, myopia, and optic disk cupping (ODC), in the leveraged fundus images. Chai et al. (2022) introduced a deep learning model using a frequent pattern mining module with an adversarial auto-encoder network. Extensive experiments were carried out on a practical image dataset to assess the performance of the integrated deep model. Instead of using the CNN-based deep learning architectures, the study Rodríguez et al. (2022) proposed a vision transformer-based model (Dosovitskiy et al., 2020) for retinal image analysis, to note that the proposed approach is similar to the study of Rodríguez et al. (2022), e.g., both of these two studies were inspired by the work of vision transformer (Dosovitskiy et al., 2020). However, there are at least the following differences between this work and ours. First of all, the input of the proposed model is image patches with linear embeddings, while Rodríguez et al. (2022) adopted CNN-based features as their input. Second, the label embeddings in the proposed model are binary while Rodríguez et al. (2022) used the ternary state embeddings in addition to the label embeddings. Originally, the transformer architecture Vaswani et al. (2017) was employed in natural language (NLP) processing applications (Galassi et al., 2019). Since the outstanding outcome of transformer yielded in NLP initially, it has been extensively employed in a variety of machine vision applications. Different from the CNN models presented in the retinal image classification, the vision transformer-based models can unveil the global associations between long-range pixels in retinal images besides the information extracted from the local receptive fields (Fang et al., 2019; Gao et al., 2022) in an image.

Bearing the above-mentioned analysis in mind, this study proposes a novel multi-label retinal image classification model inspired by the original vision transformer (Dosovitskiy et al., 2020). A publicly available retinal image dataset ODIR-2019^1^ was exploited to complete the training of the proposed approach. To evaluate the performance of the proposed transformer model, the comparison experiments were conducted using the public dataset ODIR-2019 between the state-of-the-art CNN architectures. Experimental results of the proposed approach demonstrate the superiority of the presented pipeline and the value of self-attention mechanism in retinal image classification.

The primary contributions of this study can be summarized as follows:

- A vision transformer-based multi-label retinal image classification pipeline is proposed.
- A vision transformer model designed for the task of multi-label classification was presented.
- Experimental outcome prove the potential value of the proposed model in clinical practice.

The subsequent sections of this article are outlined below. The specifics of the proposed pipeline are outlined in Section 2. Section 3 outlines the experimental methodology employed to assess the efficacy of the suggested technique. The study's discussion and conclusion are presented in Section 4.

## 2 Methodology

### 2.1 Dataset

The proposed vision transformer model was instantiated by using the public multi-label retinal image database ODIR-2019. ODIR-2019 was first provided by the Ocular Disease Intelligent Recognition (ODIR) in 2019 University International Competition. It is composed of the retinal images containing eight different types of retinal lesions in total, which are AMD (A), cataract (C), DR (D), glaucoma (G), hypertension (H), myopia (M), other abnormalities (O), and the control group of normal (N). Moreover, this dataset also contains the subject-wise labels with both the images and the medical records of the patients. Totally, 3,500 annotated retinal images from 5,000 cases were incorporated within the dataset. The details of the dataset distribution are shown in Table 1. The entire set of images were divided into training (70%), testing (20%), and validation set (10%).

**Table 1:** Detailed distribution of the ODIR-2019 dataset.

**Category**	**Full name**	**Number of images**
A	Age-related macular degeneration	171
C	Cataract	211
D	Diabetes retinopathy	1,131
G	Glaucoma	207
H	Hypertension	94
M	Myopia	177
O	Other abnormalities	944
N	Normal	1,135

**Figure 1:**
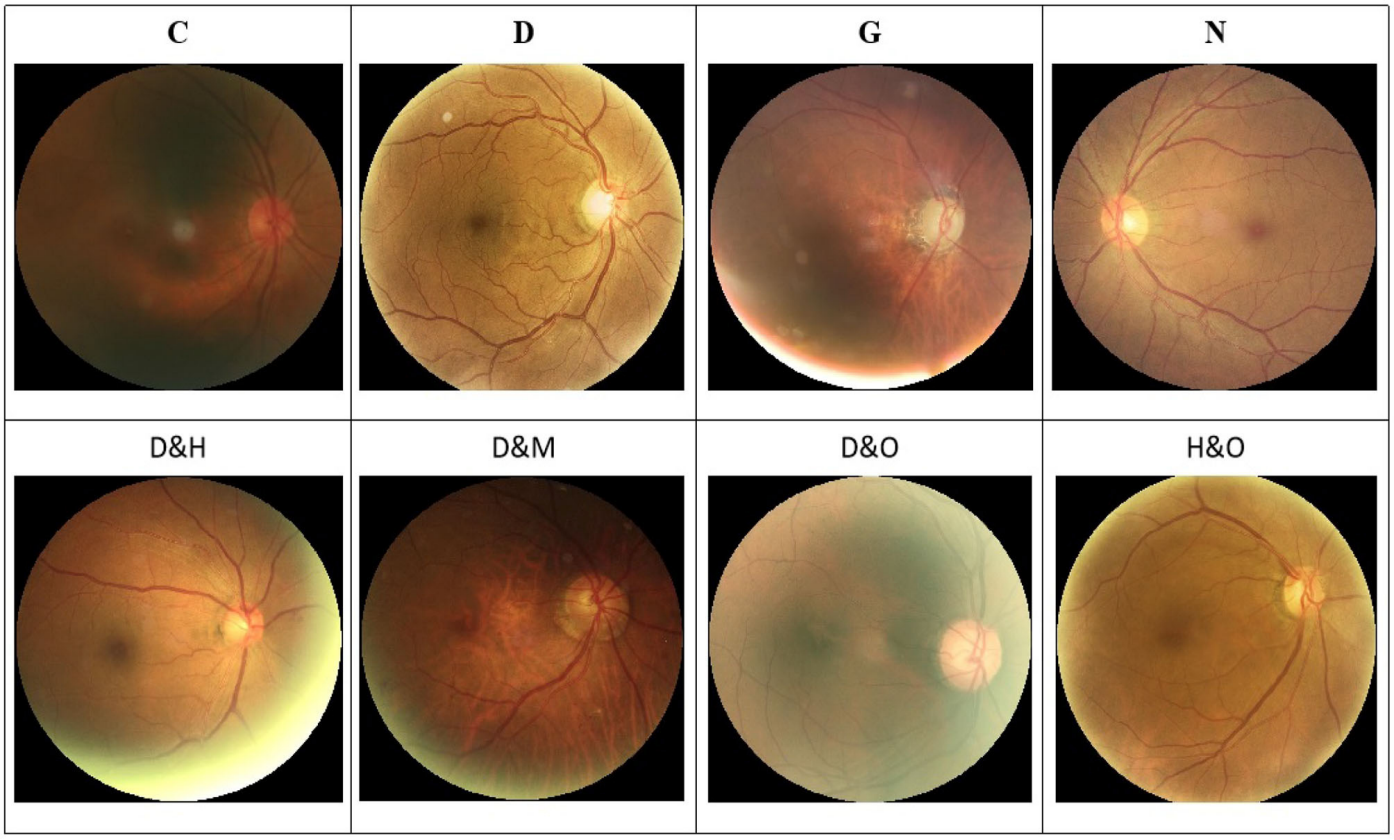
Image samples in the ODIR-2019 dataset. **(Top row)** The single-label retinal images. **(Bottom row)** The multi-label retinal images. C, D, G, H, M, N, and O denote the cataract, diabetes retinopathy, glaucoma, hypertension, myopia, and other abnormalities retinal images, respectively.

In addition, a set of samples in the ODIR-2019 dataset are provided in Figure 1. Specifically, there are both single-label and multi-label retinal images in this dataset.

### 2.2 Multi-label classification network architecture

This study aimed at addressing the multi-label classification of retinal images, which can be expressed mathematically as follows. To note that each image inside the recordings is represented by the symbol *I*_*i*_, where *i* belongs to the range [1, *N*′]. Here, *N*′ represents the total count of images present. In this study, the label of each image could be denoted as a vector $y_{j}=\left(y_{1},...,y_{N}^{{\;}^{\prime }}\right)\in {\left\{0,1\right\}}^{C^{{\;}^{\prime }}}$, where *C*′ represents the total number of retinal lesion categories. Each marking denotes the presence (1) or absence (0) of each specific retinal lesion.

**Figure 2:**
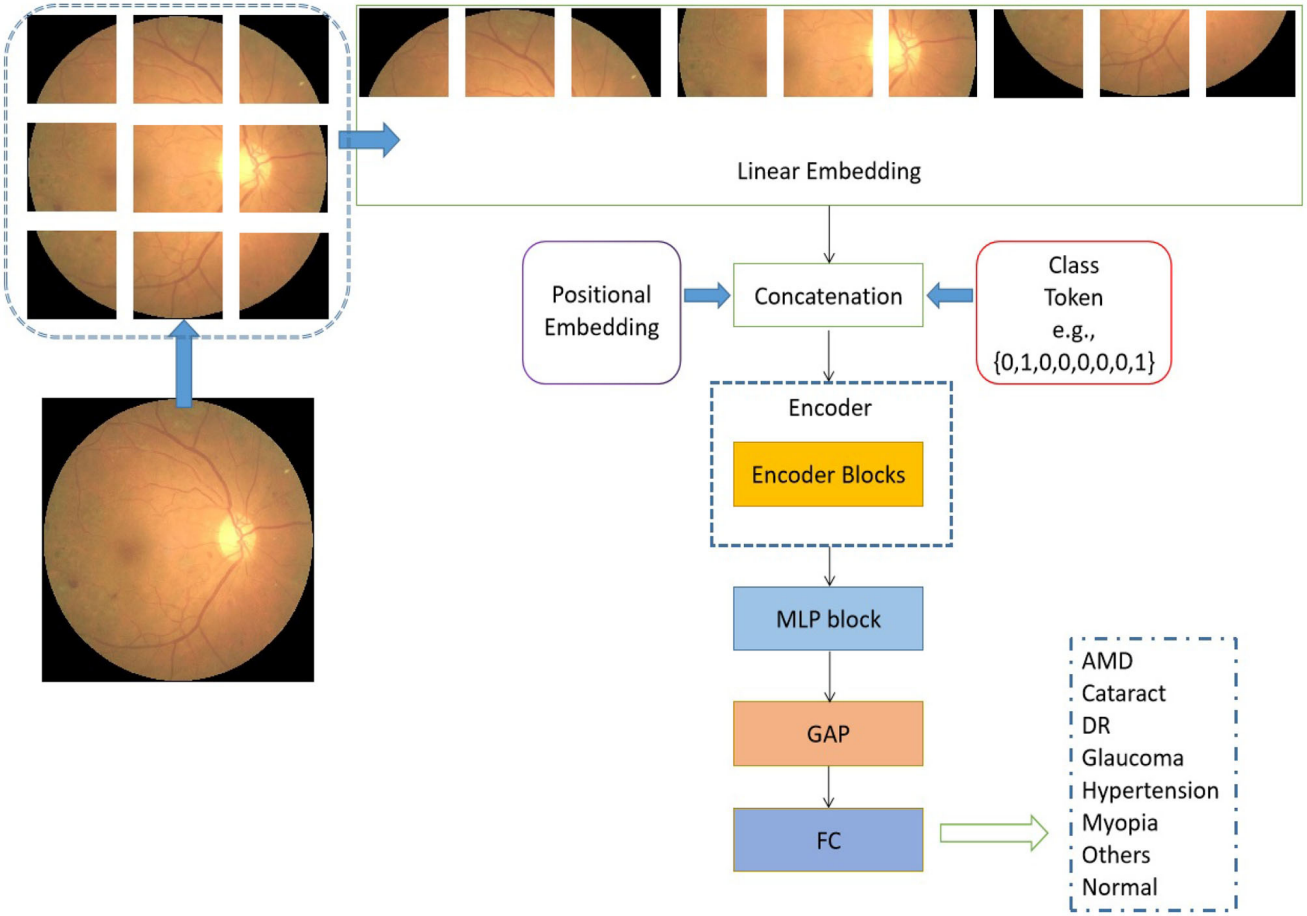
Architectural of the proposed vision transformer. L is used to represent the quantity of encoder blocks in this model.

The schematic representation of the transformer model under consideration, as seen in Figure 2, is based on the architectural design of the vision transformer (Dosovitskiy et al., 2020). The initial step involves the utilization of a retinal image as input, which is subsequently transformed into flattened linear embeddings. To handle the two-dimensional retinal images, the proposed model employs to reshape the images *I*∈*R*^*h*×*w*×*d*^ into smaller image patches $I_{p}\in R^{n\times p\times p\times d}$. It should be noted that the variable *h*×*w* = 224 × 224 is used to represent the resolution of the original image. Additionally, the variable *p*×*p* specifies the size of each image patch. The variable *d* is assigned a value of 3, which represents the number of channels in an RGB image. The variable *n* is calculated as the quotient of *h*×*w* divided by *p*×*p*. To account for the distribution of image patches inside each original image, positional embeddings are concurrently appended to the flattened embeddings (Dosovitskiy et al., 2020). The positional embedding serves the purpose of denoting the spatial position of the image patches inside an image.

In addition to the linear embedding layer, the proposed model primarily consists of two other components: an encoder block and a multiple-layer perception (MLP) module. It is important to acknowledge that each input sequence of retinal images corresponds to the types of retinal fundus lesions. In addition, the encoder block incorporates the pivotal multi-head self-attention module (Vaswani et al., 2017), which is designed to uncover the relationships among distant image pixels. Furthermore, to achieve a coherent encoder module, the suggested model employs an iterative repetition of the encoder block. In addition to the multi-head self-attention modules, the encoders also incorporate several other types of layers, including layer normalization, dropout, and MLP blocks. The purpose of employing the MLP block was to produce the output for multi-label classification by combining the global average pooling (GAP) unit (Ramasamy et al., 2021) and the fully connected (FC) layer. In a broad sense, the retrieved depiction derived from the retinal images comprises both localized information pertaining to a sequence of signals and the overarching correlation between signals that are widely separated.

**Figure 3:**
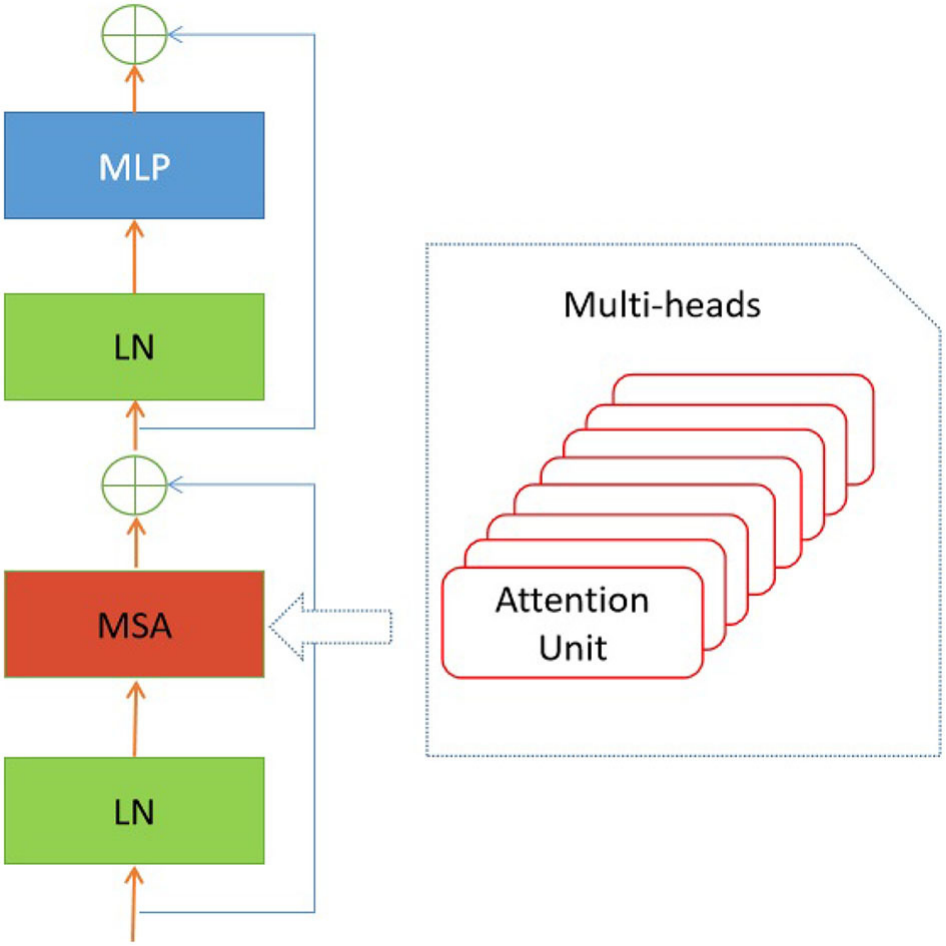
Encoder block in the presented transformer model.

In the suggested transformer model, the input sequences of retinal images undergo a sequential flattening process, resulting in the transformation of these sequences into vectors. Furthermore, it is important to acknowledge that the encoder block is iterated a variable number of times in different iterations of the proposed transformer model. Additionally, the diagram depicting the structural configuration of this encoder block can be observed in Figure 3.

**Figure 4:**
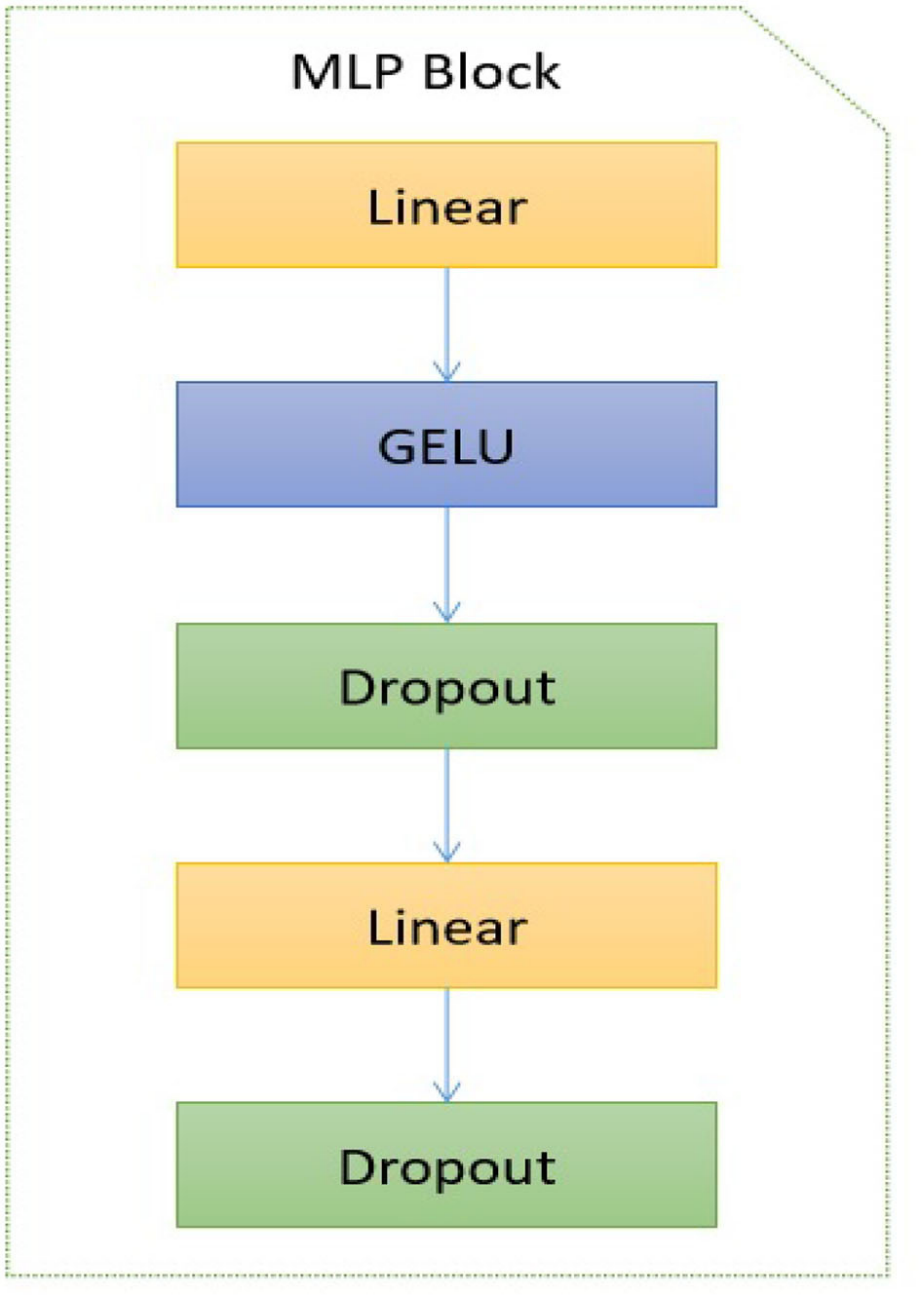
MLP block used in the proposed transformer model. GELU denotes the activation function (Lee, 2023).

As depicted in Figure 3, the encoder block comprises several distinct components, including layer normalization, multi-head self-attention (MSA), dropout, and MLP block. The study did not conduct a thorough analysis of the MSA unit as it has already been extensively studied in the current literature (e.g., Zhou et al., 2022). The study conducted by Guo and Gao (2022) employed a unit comprised of *H*′ heads to evaluate the similarity between a query and its corresponding keys, taking into account the allocated weight for each value. In addition, the layer normalization module is utilized to compute the mean and variance necessary for normalizing the inputs to the neurones within a layer during a single training instance (Ba et al., 2016). In this study, the authors employ the dropout layer (Choe and Shim, 2019) as a means of regularization to address the potential issue of over-fitting. The architectural structure of the multi-layer perceptron (MLP) block is depicted in Figure 4.

The technique that has been proposed enables the formulation of the process of categorizing retinal lesions in the following Equations (1–5):


(1) $$\begin{array}{l} z_{0}=\left[x_{class};x_{p}^{1}E;x_{p}^{2}E;...;x_{p}^{m}\right]+E_{position}, \end{array}$$

where variable *z*_0_ denotes the output of the linear embedding layer. In the present situation, the variable *m* denotes the quantity of channels employed in a linear embedding. The variables *x*_*class*_ and *E*_*position*_ correspond to the class token and positional embedding, respectively. In the context of multi-label classification, it is worth noting that the class token *x*_*class*_ utilized in the proposed model exhibits distinct characteristics compared to the single-label class token employed in the original vision transformer (Dosovitskiy et al., 2020).


(2) $$\begin{array}{l} z_{l}^{{\;}^{\prime }}=MSA\left(LN\left(z_{l-1}\right)\right)+z_{l-1}, \end{array}$$

(3) $$\begin{array}{l} z_{l}=MLP\left(LN\left(z_{l}^{{\;}^{\prime }}\right)\right)+z_{l}^{{\;}^{\prime }}, \end{array}$$

(4) $$\begin{array}{l} y=FFN\left(z_{L}^{0}\right), \end{array}$$

where layer normalization unit is represented as *LN*(.). In this notation, *z*_*l*_ represents the output of layer *l*. The feed-forward network integrated with a fully connected (FC) layer and a sigmoid activation function is written as FFN(.). The output classification outcome is denoted as *y*.

The loss function employed throughout the training procedure is the weighted binary cross entropy function:


(5) $$\begin{array}{l} Loss=-\frac{1}{M}\overset{C^{{\;}^{\prime }}}{\underset{c=1}{\sum }}y_{i}log\left(p\left(y_{c}\right)\right)+\left(1-y_{c}\right)log\left(1-p\left(y_{c}\right)\right), \end{array}$$

where *C* denotes the number of retinal lesion categories.

## 3 Experiments

### 3.1 Implementation details

The transformer model described in this study is implemented utilizing the PyTorch framework (Paszke et al., 2019). The system utilizes four NVidia RTX 3090 Graphical Processing Units (GPUs) with a combined RAM capacity of 128GB for computing purposes. The optimal parameters of the proposed network are determined through a trial and error methodology. A 10-fold cross-validation approach is utilized to evaluate the reliability and stability of the proposed methodology. The other implementation details are provided in Table 2. Then, the retinal data input was divided into ten equally sized groups in a sequential manner. In each iteration, a single group out of the total of ten was assigned the role of the testing set, while the remaining nine groups were employed as the training set. Ultimately, the final output is determined by utilizing the mean result obtained from 10 iterations.

**Table 2:** Implementation details in the experiments.

**Item**	**Value**
Batch_size	8
Optimizer	Adam
Learning rate	1e-4
Weight decay	0.02
Epochs	100

### 3.2 Evaluation metrics

In addition, the evaluation metrics included in the trials included the F1 score, Kappa coefficient, AUC, and the average of these three performance indicators. The mathematical representation of these metrics is explicated in the subsequent equations:

(1) The definition of Kappa is provided in Equations (6, 7, and 8).


(6) $$\begin{array}{l} kappa=\frac{p_{o}-p_{e}}{1-p_{e}}, \end{array}$$

(7) $$p_{o}=\frac{\sum_{c=1}^{C}TP_{c}}{\sum_{c=1}^{C}\left(TP_{c}+FN_{c}\right)},$$

(8) $$p_{e}=\frac{\sum_{c=1}^{C}TP_{c}\times (TP_{c}+FN_{c})}{N\times N},$$

where the phrases true positive and false negative are denoted as TP and FN, respectively. The variable *c* represents the number of retinal lesion categories, whereas *N* represents the total number of image samples.

(2) The used F1 score is expressed as Equations (9, 10, and 11).


(9) $$\begin{array}{l} F1=2\times \frac{Precision\;\times \;Recall}{Precision\;+\;Recall}=2\times \frac{TP}{2\;\times \;TP\;+\;FN\;+\;FP}, \end{array}$$

(10) $$\begin{array}{l} Precision=\frac{TP}{TP\;+\;FP}, \end{array}$$

(11) $$\begin{array}{l} Recall=\frac{TP}{TP\;+\;FN}, \end{array}$$

where the terms FP and FN represent false positive and false negative, respectively.

(3) AUC is given in Equations (12, 13, and 14).


(12) $$AUC=\int_{x=0}^{1}TPR(FPR^{-1}(x))dx,$$

(13) $$\begin{array}{l} TPR=\frac{TP}{TP\;+\;FN}, \end{array}$$

(14) $$\begin{array}{l} FPR=\frac{FP}{FP\;+\;TN}. \end{array}$$

### 3.3 Ablation study

To ascertain the most suitable architecture for the proposed vision transformer, a comprehensive evaluation was conducted to determine the optimal combination of the hyper-parameters used in the proposed model. In the ablation study, we considered the number of encoder blocks (*L*) in the encoder, as depicted in Figure 3, and the number of MSA heads (*H*′) employed in a single encoder block, as demonstrated in Figure 3.

The in-depth findings of the ablation study can be found in Table 3. It is important to keep in mind that only 10% of the retinal images were used in the study that involved ablation. In the meantime, the area under the curve (AUC) was used as the evaluation statistic for this algorithm.

**Table 3:** Combinations of *L* and *H* and the comparison performance of the proposed model with these combinations.

**Model**	**Number of layers (*L*)**	**Number of heads (*H*^′^)**	**AUC**
L_2_H_8	2	8	0.907
L_4_H_8	4	8	0.911
L_8_H_8	8	8	0.923
L_2_H_16	2	16	0.917
L_4_H_16	4	16	**0.931**
L_8_H_16	8	16	0.925

L, number of layers; H, number of heads.

The most effective combination of *L* and *H*′ may be determined by referring to Table 3. Specifically, the combination of *L* = 4 and *H* = 16 demonstrates optimal results. This combination is subsequently utilized in the subsequent experiments conducted for the suggested approach.

### 3.4 Performance of the proposed method and the comparison experiments

**Figure 5:**
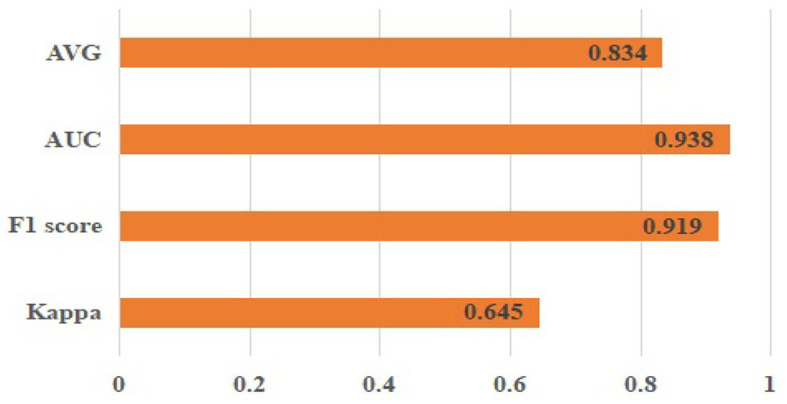
Classification results of the proposed approach on the ODIR-2019 dataset.

This section first presents the outcomes obtained by implementing the proposed methodology on the publicly accessible dataset ODIR-2019. The classification results are presented in Figure 5. The corresponding outcomes are Kappa (0.645 ± 0.04), F1 score (0.919 ± 0.02), AUC (0.938 ± 0.05), and AVG ($AVG=\frac{Kappa+F1+AUC}{3}$, 0.834 ± 0.04).

**Figure 6:**
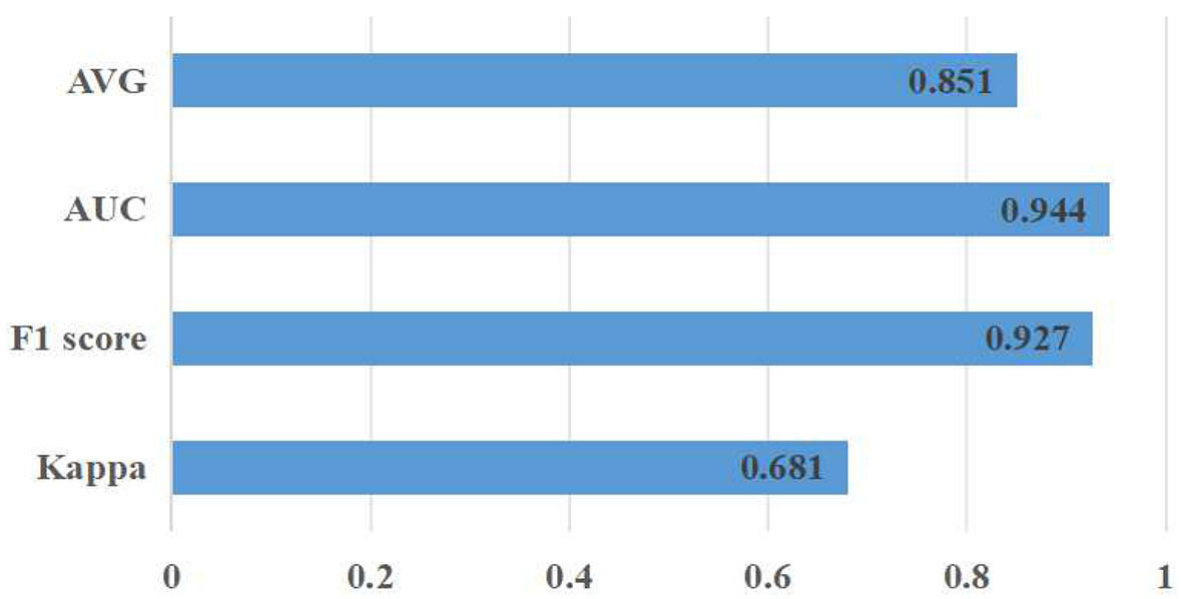
Classification results of the proposed approach on the RFMiD 2.0 dataset.

Meanwhile, a hold-out test was conducted to evaluate the proposed approach on entirely new data, which had not been used in the training process. Thus, the RFMiD 2.0 data (Panchal et al., 2023) were exploited in the hold-out test Figure 6. The corresponding experimental results are Kappa (0.681 ± 0.03), F1 score (0.927 ± 0.04), AUC (0.944 ± 0.08), and AVG (0.851 ± 0.03).

**Figure 7:**
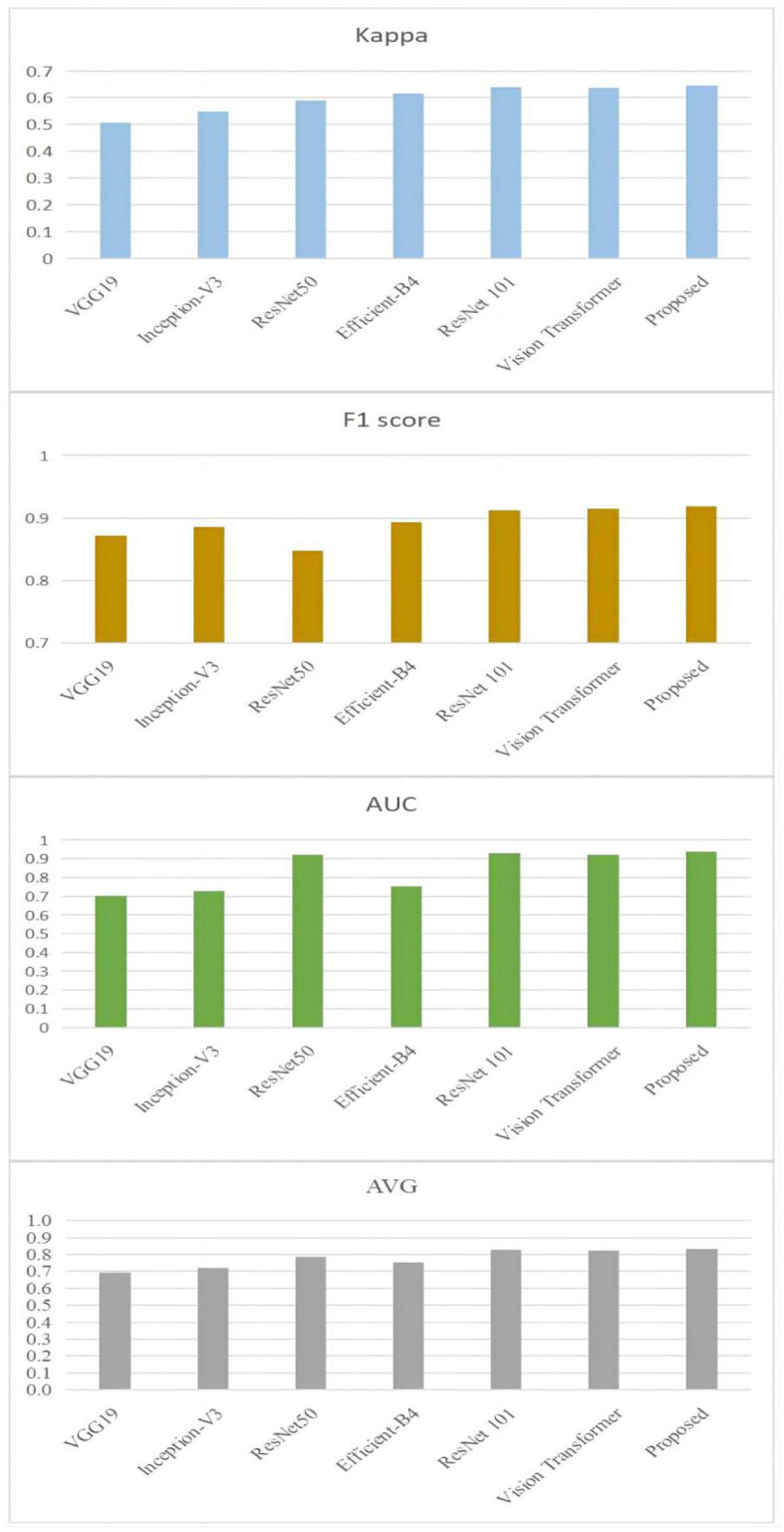
Comparison results between the proposed approach and the state-of-the-art techniques on the ODIR-2019 dataset.

In order to provide further evidence of the effectiveness of the provided approach, experiments comparing our model to the most recent and cutting-edge CNN models have been carried out. Models such as VGG19 (Simonyan and Zisserman, 2014), ResNet50 (He et al., 2015), Inception-V3 (Szegedy et al., 2014), Efficient-B4 (Tan and Le, 2019), ResNet101 (He et al., 2015), and vision transformer (Rodríguez et al., 2022) are considered to be among the most advanced currently available. The results of the comparison are presented in Figure 7.

**Figure 8:**
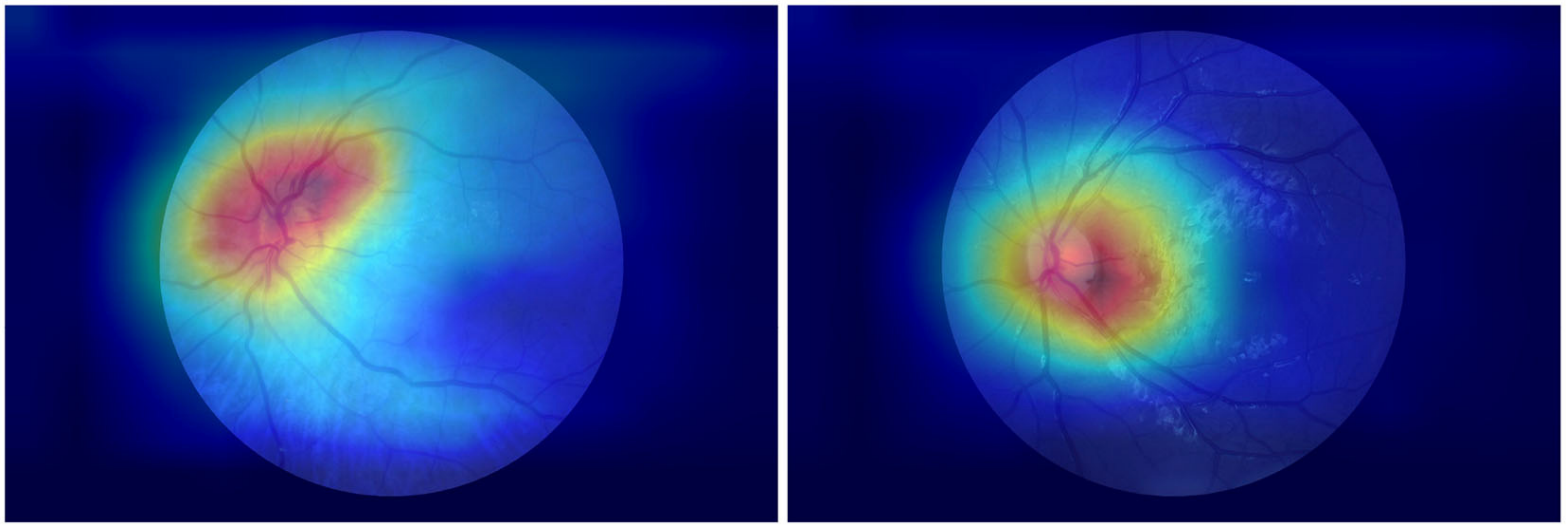
CAMs generated using the proposed approach.

Furthermore, the class activation mapping (CAM) figures generated by using the proposed approach with the public dataset are provided in Figure 8.

Finally, to evaluate the proposed model in classifying each category of retinal diseases, the single-label classification experiment was conducted by the proposed approach on the ODIR-2019 dataset. The corresponding results are F1 score (0.932 ± 0.06) and AUC (0.950 ± 0.03).

### 3.5 Discussion

It is clear by looking at Figure 7 that the proposed methodology has reached a higher level of performance when compared to the ways that are currently being used. To be more specific, the Kappa value of the technique that is being proposed is 0.645. It has increased by 9.38 % in comparison with the one that was produced by ResNet101's work (He et al., 2015), which was the closest one. In addition, in comparison with the one that was created by ResNet101, the F1 score of the suggested approach has grown by 7.68 %, the value of the approach's AUC has increased by 0.97 %, and the approach's average value has increased by 0.85 %.

There are also several limitations need to be mentioned in this study. First of all, this study did not take the imbalanced issue existed in the leveraged dataset into consideration. In the ODIR-2019 dataset, there are much more images in the DR (D), normal (N), and other abnormalities (O) categories than the remaining five classes. Therefore, the imbalanced distribution of the dataset might have an influence on the performance of the proposed approach. Second, the presented deep model was inspired by the original vision transformer (Dosovitskiy et al., 2020), and the primary modification to the original vision transformer mainly locates at the output layer to adapt to the requirement of multi-label classification. The inner structure of the vision transformer needs should also be optimized to yield a more accurate result. Finally, only one specific dataset was exploited in the experiments, which might not be able to prove the generalization of the proposed vision transformer architecture.

## 4 Conclusion

In this study, a novel vision transformer model was presented to resolve the multi-label retinal image classification issue. In total, eight categories of retinal images can be classified by the proposed approach. Experimental results demonstrate the superiority of our method over the state-of-the-art CNN-based models. To note that it can be attributed to the leveraged attention mechanism in the proposed deep learning model, which is supposed to reveal the global associations between long-range pixels.

In the future, more data samples will be incorporated to enhance both the diversity of the images and the generalization of the model presented in this study. In addition, a variety of the combinations of CNN and transformer modules would be exploited to develop more optimal deep models.

## Data availability statement

The original contributions presented in the study are included in the article/supplementary material, further inquiries can be directed to the corresponding authors.

## Author contributions

DW: Writing – original draft. JL: Writing – original draft. WJ: Writing – original draft.

## Footnotes

^1^ https://odir2019.grand-challenge.org
